# Neurovascular effects of tadalafil in patients with cerebral small vessel disease: ETLAS-2 substudy

**DOI:** 10.1093/braincomms/fcag309

**Published:** 2026-08-13

**Authors:** Joakim Ölmestig, Kristian N Mortensen, Birgitte Fagerlund, Nadia Naveed, Mette M Nordling, Hanne Christensen, Helle K Iversen, Mai B Poulsen, Hartwig R Siebner, Christina Kruuse

**Affiliations:** Neurovascular Research Unit, Department of Neurology, Copenhagen University Hospital—Herlev and Gentofte, Herlev 2730, Denmark; Danish Research Centre for Magnetic Resonance, Department of Radiology and Nuclear Medicine, Copenhagen University Hospital—Amager and Hvidovre, Hvidovre 2650, Denmark; Department of Clinical Medicine, University of Copenhagen, Copenhagen 2200, Denmark; Danish Research Centre for Magnetic Resonance, Department of Radiology and Nuclear Medicine, Copenhagen University Hospital—Amager and Hvidovre, Hvidovre 2650, Denmark; Child and Adolescent Mental Health Center, Copenhagen University Hospital, Mental Health Services CPH, Hellerup 2900, Denmark; Department of Psychology, University of Copenhagen, Copenhagen 1353, Denmark; Department of Radiology, Copenhagen University Hospital—Herlev and Gentofte, Herlev 2730, Denmark; Department of Radiology, Copenhagen University Hospital—Herlev and Gentofte, Herlev 2730, Denmark; Department of Clinical Medicine, University of Copenhagen, Copenhagen 2200, Denmark; Department of Neurology, Copenhagen University Hospital—Bispebjerg and Frederiksberg, Copenhagen 2400, Denmark; Department of Clinical Medicine, University of Copenhagen, Copenhagen 2200, Denmark; Department of Neurology, Copenhagen University Hospital—Rigshospitalet, Glostrup 2600, Denmark; Department of Neurology, Copenhagen University Hospital—North Zealand, Hillerød 3400, Denmark; Danish Research Centre for Magnetic Resonance, Department of Radiology and Nuclear Medicine, Copenhagen University Hospital—Amager and Hvidovre, Hvidovre 2650, Denmark; Department of Clinical Medicine, University of Copenhagen, Copenhagen 2200, Denmark; Department of Neurology, Copenhagen University Hospital—Bispebjerg and Frederiksberg, Copenhagen 2400, Denmark; Neurovascular Research Unit, Department of Neurology, Copenhagen University Hospital—Herlev and Gentofte, Herlev 2730, Denmark; Department of Clinical Medicine, University of Copenhagen, Copenhagen 2200, Denmark; Department of Brain and Spinal Cord Injury, Neuroscience Centre, Copenhagen University Hospital—Rigshospitalet, Glostrup 2600, Denmark

**Keywords:** cerebral blood flow, cerebrovascular reactivity, neurovascular coupling, pCASL, fMRI

## Abstract

Cerebral small vessel disease is a major contributor to stroke and cognitive impairment, often associated with reduced cerebral blood flow and cerebrovascular reactivity. In this study, we used MRI to investigate if daily treatment with the phosphodiesterase-5 inhibitor tadalafil over 3 months improved cerebral blood flow and cerebrovascular reactivity in patients with cerebral small vessel disease.

Using a randomized, placebo-controlled, parallel-group design, this MRI sub-study was prospectively performed as part of the Effect of Tadalafil in Lacunar Stroke 2 trial, which included a total of 76 participants, of whom 60 were included in the per-protocol analysis. Patients with cerebral small vessel disease and prior stroke or transient ischaemic attack received either tadalafil (20 mg/day) or placebo for 3 months and completed the study. Neurovascular outcomes were assessed at baseline and at the 3-month follow-up using dual-echo pseudo-continuous arterial spin labelling MRI to evaluate reactivity to hypercapnia and visual stimulation. Endpoints included changes in whole-brain baseline cerebral blood flow and cerebrovascular reactivity, visual stimulation response, and neurovascular coupling index. In addition, we investigated arterial transit time measured using diffusion-prepared pseudo-continuous arterial spin labelling and changes in event-related sensory blood-oxygen-level-dependent responses in the primary somatosensory cortex (S1) after stimulation of the index finger.

In total, 60 participants (median age: 66.5, 14 females) were included in this per-protocol sub-study analysis (28 on tadalafil and 32 on placebo). Forty-two pseudo-continuous arterial spin labelling datasets (20 on tadalafil and 22 on placebo), 53 blood-oxygen-level-dependent functional MRI datasets (26 on tadalafil and 27 on placebo), and 45 arterial transit time datasets (22 on tadalafil and 23 on placebo) were of sufficient quality to be included in the analysis. Baseline whole-brain cerebral blood flow was 25.4 ± 5.1 ml/100 g/min. Compared to placebo, tadalafil significantly increased whole-brain cerebral blood flow with a mean group difference of 3.33 ± 0.97 ml/100 g/min at follow-up (*P* = 0.001). None of the other MRI-based outcome metrics differed between groups.

Three months of tadalafil treatment significantly enhanced whole-brain cerebral blood flow in patients with cerebral small vessel disease without altering cerebrovascular reactivity, neurovascular coupling, arterial transit time, and event-related blood-oxygen-level-dependent responses. The clinical significance of a tadalafil-induced increase in cerebral blood flow remains to be determined in prospective studies covering longer treatment periods.

## Teaser

Ölmestig *et al*. studied whether daily tadalafil could improve brain blood flow in people with stroke and small vessel disease. It increased overall brain blood flow but not the response during brain activity. Tadalafil may aid brain circulation, but its clinical benefit needs further research.

## Graphical Abstract

**Graphical Abstract:**
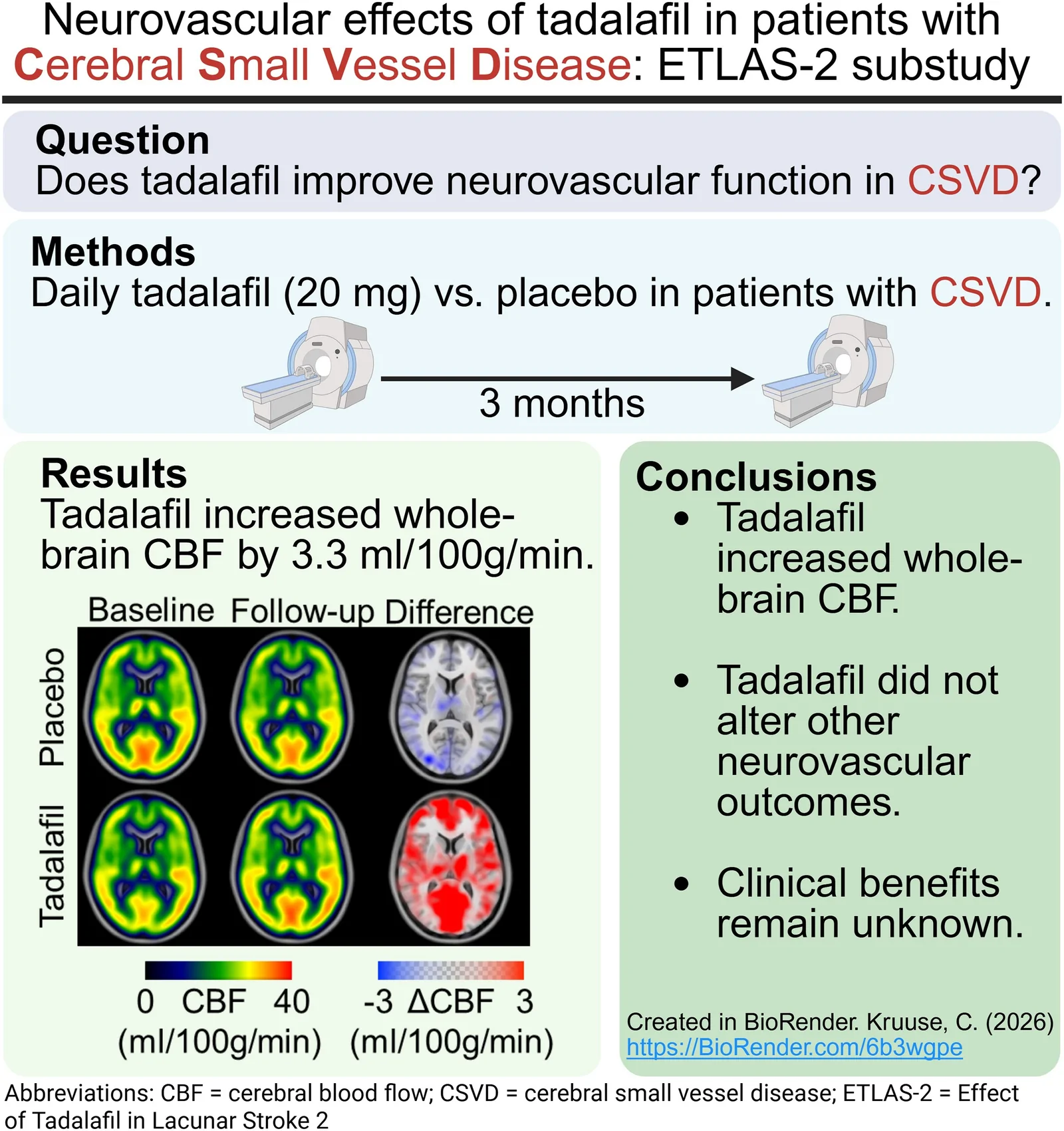

## Introduction

Cerebral small vessel disease (CSVD) is a cerebrovascular disorder characterized by impaired vascular function and reduced cerebral blood flow (CBF).^1^ CSVD is a major contributor to ischaemic and haemorrhagic stroke, cognitive impairment, and vascular dementia, representing a substantial burden on patients and the healthcare systems.^2^ There is currently no specific treatment to reduce the impact of CSVD and improve cerebrovascular function. Vasoactive compounds have been proposed for use in CSVD, which targets the cyclic nucleotide signalling in cerebral vessels. Recently, the OxHARP trial found improved CBF and cerebrovascular reactivity (CVR) with daily dosing of sildenafil in patients with CSVD.^3^

White matter hyperintensity (WMH) visualized on magnetic resonance imaging (MRI) is a hallmark of CSVD.^4^ A systematic review with meta-analysis showed an association between reduced CBF and a high burden of WMH.^1,5^ In addition to reduced CBF, CVR may also be impaired.^6,7^ Nonetheless, the pathophysiological mechanisms and clinical symptoms remain to be fully established.^8^ The role of endothelial dysfunction in CSVD has gained increasing attention and is likely to play a crucial role in the pathophysiology of CSVD.^8,9^ Reduced nitric oxide (NO)—cyclic guanosine monophosphate (cGMP), an important molecular pathway in endothelial-induced vascular reactivity, can be pharmacologically enhanced by phosphodiesterase 5 (PDE-5) inhibitors. We propose that PDE-5 inhibitors may specifically enhance cerebrovascular function in patients with CSVD and represent a disease-modifying treatment to reduce the clinical burden of CSVD.

In this paper, we report the neurovascular MRI findings from the Effect of Tadalafil in Lacunar Stroke 2 (ETLAS-2) Trial, evaluating the effects of daily tadalafil (20 mg) compared to placebo in patients with a history of stroke and imaging signs of CSVD. Clinical and cognitive outcomes from the ETLAS-2 trial are reported separately.^10,11^ Here, we specifically investigated if tadalafil improved cerebrovascular function, including CBF, CVR, and neurovascular coupling (NVC). The results presented in this paper are hypothesis-generating for future studies on disease-modifying treatment of CSVD.

## Materials and methods

### Trial design

We conducted the ETLAS-2 trial as a randomized, placebo-controlled, double-blind, parallel study to test the feasibility of daily oral tadalafil 20 mg against placebo for 3 months and its effect on neurovascular function and cognition, as specified in the published trial protocol.^12^ The study was pre-registered at Clinicaltrials.gov (NCT05173896) and approved by the Research Ethics Committee in the Capital Region of Denmark (H-20031301), the Danish Medicines Agency (EudraCT: 2020-002329-27), and the Capital Region at Knowledge Centre for Data Reviews (P-2020-1193). Consent was obtained from all participants in accordance with the Declaration of Helsinki. The Good Clinical Practice Unit in Denmark monitored the study. We enrolled patients from four neurological departments in the Capital Region of Denmark: Copenhagen University Hospital—Herlev and Gentofte (HGH); Copenhagen University Hospital—Rigshospitalet (RH); Copenhagen University Hospital—Bispebjerg and Frederiksberg (BFH); and Copenhagen University Hospital—North Zealand (NZH). MRI scans were performed at the Danish Research Centre for Magnetic Resonance (DRCMR), Copenhagen University Hospital—Amager and Hvidovre (AHH). This manuscript follows the CONSORT guidelines.

### Study visits and participants

**Figure 1:**
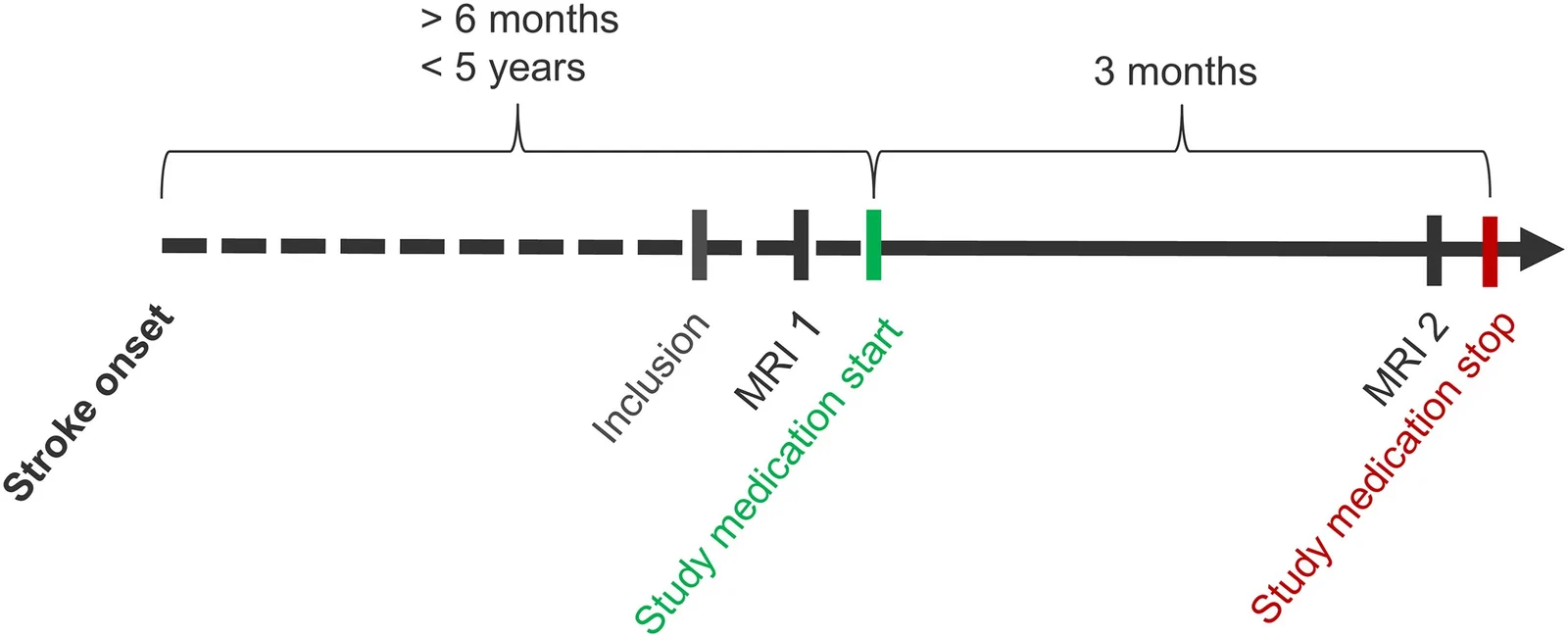
**Study flow chart with the timing of MRI.** The flow chart was adapted from the original article (with permission from *BMC*, *Copyright* under Creative Commons Attribution 4.0 International License: http://creativecommons.org/licenses/by/4.0/)^12^.

We included patients above the age of 50 years who had been hospitalized between 6 months and 5 years prior to study inclusion with a stroke or transient ischaemic attack (TIA) event and with signs of CSVD on brain imaging performed at hospital admission. CSVD imaging signs included small vessel occlusion infarct (≤2 cm diameter in the acute phase and ≤1.5 cm diameter in the late phase) and/or white matter hyperintensity (WMH) graded ≥2 on a combined deep and periventricular Fazekas scale.^12^ Acute stroke patients were not included since administration of tadalafil is currently contraindicated within 6 months of a stroke event, and our aim was not to investigate the effect of tadalafil on the ischaemic event but the underlying pathology of CSVD. All included patients provided written and oral informed consent before inclusion in the trial, and randomization was performed in variable block sizes by the Capital Region Pharmacy, which packed trial medication in a 1:1 ratio between tadalafil and placebo in opaque capsules to ensure blinding of the treatment. During inclusion, participants were given study medication corresponding to their unique randomization number.^12^ We conducted the baseline MRI prior to the participant commencing the study medication and again at the end of the study period (3 months) while the participant was still taking the study medication (Fig. 1). A full description of inclusion and exclusion criteria, together with study visits, has been published.^12^

### MRI

Brain MRI was conducted at baseline and after 3 months on a Philips 3 Tesla Achieva TX scanner (Philips, The Netherlands). MRI acquisition and analyses were performed blinded to the allocated treatment. We acquired dual-echo pseudo-continuous arterial spin labelling (pCASL) to evaluate whole-brain CBF, CVR, and NVC. We assessed event-related blood-oxygen-level-dependent (BOLD) functional MRI (fMRI) during sensory stimulation of the dominant index finger and acquired diffusion-prepared pCASL (DP-pCASL) for evaluation of arterial transit time (ATT) and blood-brain barrier (BBB) water exchange rate. Structural MRI included T1w, T2w, fluid-attenuated inversion recovery (FLAIR), diffusion-weighted imaging (DWI), susceptibility-weighted imaging (SWI), and time-of-flight angiography (TOF). Structural MRI data are reported in the ETLAS-2 main study paper^10,^ and details of the acquisition parameters are reported previously.^12^ All dual-echo pCASL scans were individually quality-checked for ATT artefacts by a neuroradiologist not otherwise involved in the study. ATT artefacts were graded as none, moderate, or severe, and participants with severe ATT artefacts were excluded from the analysis.

### Dual-echo pCASL MRI

We performed dual-echo pCASL MRI during normo- and hypercapnia (5% CO_2_, 20% O_2_, in N_2_) with visual stimulation.^13^ It was performed in five blocks of 4 min and 48 s, alternating between normocapnia (first, third, and fifth blocks) and hypercapnia (second and fourth blocks). During the second half of each block, two visual stimulations of 36 s each were given, separated by a 36 s pause. Visual stimulation consisted of on/off contrast-reversing checkerboard stimulation programmed in the PsychoPy 2 software, automatically triggered from the MRI software. Imaging consisted of a pCASL sequence (post-labelling delay (PLD): 2000ms, labelling duration: 1650 ms, labelling plane positioned at a 90-degree angle to the internal carotid artery superior to the carotid bulb) with a dual echo, 2D echo planar imaging (EPI) read-out (echo times (TE): 10/30 ms, repetition time (TR): 4.5 s, global background suppression pulses: 1680/3150 ms, in-plane resolution: 3.75 × 3.75 mm, matrix: 64 × 64, 14 slices acquired in ascending order, slice thickness: 7 mm, SENSE factor: 2.0 (AP), EPI factor: 63). For quantification CBF, an equilibrium magnetization reference image (M0-image) was acquired using the same parameters but with TR: 6 s and no background suppression.

### BOLD fMRI

We assessed event-related BOLD response during electrical sensory stimulation of the dominant index finger, as defined by the Edinburgh Handedness Scale. The scan lasted 5 min and 8 s. Stimulation was given by a Digitimer DS5 stimulator (Digitimer Ltd, UK). Before each BOLD fMRI scan, the sensory threshold was measured and defined as the limit to which the participant could sensate the electrical stimulation. The stimulations were set to three times the sensory threshold during the BOLD fMRI sequence. Stimulations were repeated 10 times as 20 pulse cycles with a 5 Hz frequency and 200 µs cycle duration, resulting in approximately 4 s of stimulation and a 30 s pause before the next set of stimulations.

### Diffusion-prepared pCASL MRI

To estimate ATT, we acquired a flow encoding arterial spin tagging (FEAST) data set during rest.^14^ Imaging consisted of a pCASL sequence (PLD: 900 ms, labelling duration: 1650 ms) with a diffusion preparation module (*b* = 0 or 14 s/mm^2^) immediately before the 2D EPI readout (same parameters as dual echo pCASL, except TE: 19 ms, TR: 4.4 s, no SENSE acceleration). To estimate the BBB water exchange rate (*k*_w_),^15^ we acquired a second set of diffusion-prepared pCASL, consisting of pCASL (PLD: 2000ms, labelling duration: 1650 ms) with a diffusion preparation module (*b* = 0 or 50 s/mm^2^) immediately before the 2D EPI readout (same parameters as dual echo pCASL, except TE: 18 ms, TR: 5.6 s, no SENSE acceleration). An M0 image was also acquired with the same parameters except TE: 18 ms, TR: 5.9 s, and no background suppression.

### MRI analysis

#### Preprocessing

pCASL image series were motion corrected and co-registered to their respective M0-images. M0-images were co-registered to anatomical images and anatomical images were affinely registered to the Montreal Neurological Institute (MNI) template. The transformations were concatenated and pCASL and M0-images were re-sliced in MNI-space with a 2 mm isotropic resolution.

#### BOLD fMRI

fMRI BOLD was analysed using the FSL FEAT pipeline. Images were motion corrected, brain extracted, smoothed with an 8 mm FWHM kernel, high-pass temporal filtered, and affinely registered to the MNI template space. We fitted a voxel-wise general linear model (GLM) to the BOLD data. The design matrix included motion parameters and sensory stimulation convolved by a haemodynamic response function (HRF) and its temporal derivatives. Average BOLD activation was calculated using a one-sample *T*-test. Second-level analyses were carried out to estimate the average BOLD activation and treatment effects.

#### Dual-echo pCASL MRI

We fitted a voxel-wise GLM to the unsubtracted data for each echo separately. The design matrix consisted of regressors for: visual stimulation; hypercapnia; ASL (0 for control and −1 for label); ASL effect of visual stimulation (visual stimulation and ASL regressors multiplied); ASL effect of hypercapnia (hypercapnia and ASL regressors multiplied); motion parameters; and low-frequency drift (cosine, high pass: 0.001 Hz). Separate regressors were used for visual stimulation during normocapnia and hypercapnia, and the regressors were convolved with HRF specific to BOLD or ASL, respectively. For hypercapnia, a custom HRF (Gamma, delay: 1.5 s, dispersion: 33 s) was used to match the timing of the hypercapnia response observed in the average patient time series. This resulted in estimates of ASL and BOLD baselines and signal changes for visual stimulation and hypercapnia. In the following, ASL and BOLD estimates were measured from the first and second pCASL echoes, respectively.

To quantify CBF baseline and signals (in units of ml/100 g/min) from ASL estimates (ΔM), we applied the following equation:^14^


(1) $$\Delta M=\;\frac{2M_{0}f\alpha }{\lambda R_{1a}}(e^{\text{-}\omega R_{1a}}\text{-}e^{\text{-}\left(\tau +\omega \right)R_{1a}}),$$

where $M_{0}$ is the M0 proton density image, *f* is CBF, *α* is the labelling efficiency (0.95), $R_{1a}$ is the longitudinal relaxation rate in blood (0.601 s^−1^), λ is the partition coefficient of water in the brain (0.9), *ω* is the post-labelling delay (slice time corrected), and *τ* is the labelling duration. Whole-brain baseline CBF and CVR (measured as CBF and BOLD response during hypercapnia) were calculated from the GLM-estimates. Due to the equipment measuring end-tidal carbon dioxide (etCO_2_) being malfunctioning, etCO_2_ values were not consistent across the patients and CVR measures were therefore not normalized to etCO_2_-values but rather reported as percent signal change.

To calibrate the BOLD and CBF signals to estimate neurovascular parameters, we applied the Davis model:^13^


(2) $$\frac{\Delta \mathrm{BOLD}}{\mathrm{BOL}D_{0}}=M\left(1\text{-}{\left(\frac{\mathrm{CMR}O_{2}}{\mathrm{CMR}O_{2|0}}\right)}^{\beta }{\left(\frac{\mathrm{CBF}}{\mathrm{CB}F_{0}}\right)}^{\alpha \text{-}\beta }\right),$$

where subscript 0 indicates baseline, i.e. no visual stimulation or hypercapnia, *α* and *β* are model parameters (here, *α* = 0.38 and *β* = 1.3), and M is a BOLD scaling parameter.

We first created a mask of voxels in the occipital cortex that showed significant increases in CBF (*t*-stat ≥ 5.1) by visual stimulation during normocapnia. We did not use BOLD responses to avoid draining veins. We then measured BOLD and CBF at baseline and increases due to visual stimulation and hypercapnia in the activation mask. M was calculated by applying Eq. 2 to hypercapnia measurements, assuming no increase in $\mathrm{CMR}O_{2}$ due to hypercapnia. $\Delta \mathrm{CMR}O_{2}=\frac{\mathrm{CMR}O_{2}}{\mathrm{CMR}O_{2|0}}-1$ was then estimated by applying Eq. 2 to visual stimulation measurements and the previously calculated value for M. A neurovascular coupling parameter (NVC) was then calculated as the ratio of visual stimulation induced ΔCBF and ΔCMRO_2_.^16^

#### Diffusion-prepared pCASL

We measured the average ASL signal (ΔM) for each of the time series (PLD: 900, *b*: 0/14 s/mm^2^ and PLD: 2000, *b*: 0/50 s/mm^2^) using a voxel-wise GLM with regressors for ASL (−1 for label, 0 for control) and motion parameters. The GLMs were fitted using a Huber regressor, which is robust to outliers, here caused by subject motion in combination with diffusion preparation.

We derived voxel-by-voxel ATT from DP-pCASL (PLD: 900 ms, b: 0/14 s/mm^2^) using the FEAST model,^14^ where Eq. 1 describes the average ASL signal with *b* = 0 s/mm^2^ and the average ASL signal with *b* = 14 s/mm^2^ is described by:


(3) $$\Delta M^{\prime }=\frac{2M_{0}f\alpha }{\lambda R_{1a}}(e^{\text{-}\delta R_{1a}}\text{-}e^{\text{-}\left(\tau +\omega \right)R_{1a}}),$$

where *δ* is ATT. Voxels with undefined results from Eq. 3 were excluded from the analysis.

BBB water exchange (*k_w_*) was calculated from DP-pCASL (PLD: 2000ms, *b*: 0/50 s/mm^2^) using a model of the ASL signal in the capillary ($\Delta M_{c}$) and brain tissue ($\Delta M_{b}$) compartments:^15^


(4) $$\Delta M_{c}=\frac{2M_{0}f\alpha }{\lambda \beta_{1}}e^{\text{-}(R_{1a}-\beta_{1})\delta }(e^{\text{-}\omega \beta_{1}}-e^{\text{-}\left(\tau +\omega \right)\beta_{1}})$$

(5) $$\begin{array}{rl} \Delta M_{b} & =\frac{2M_{0}f\beta_{2}}{\lambda }[\frac{e^{\text{-}(R_{1a}-R_{1b})\delta }}{R_{1b}}(e^{\text{-}\omega R_{1b}}-e^{\text{-}\left(\tau +\omega \right)R_{1b}})\\ & \;-\;\frac{e^{\text{-}(R_{1a}-\beta_{1})\delta }}{\beta_{1}}(e^{\text{-}\omega \beta_{1}}-e^{\text{-}\left(\tau +\omega \right)\beta_{1}})] \end{array}$$

where $R_{1b}$ is the longitudinal relaxation rate in blood (0.7 s^−1^), $\beta_{1}=k_{w}+R_{1a}$, $\beta_{2}=\frac{k_{w}}{k_{w}+R_{1a}-R_{1b}}$, and *δ* is the voxel-wise ATT-parameter calculated above.

### Sample-size calculation

The sample size calculation was based on the primary outcome of the ETLAS-2 main study. It stated that 64 participants were needed for the primary outcome (treatment feasibility). A second sample size calculation used 3-Tesla ASL MRI data from healthy individuals, as limited 3-Tesla ASL data in patients with CSVD were available during study design. It showed that we needed a total of 50 participants to identify a treatment effect of 15% with a statistical power of 80% on CBF in grey matter, 58 participants for white matter, and 72 participants for deep grey nuclei. Therefore, we aimed to include 100 participants to account for dropouts.^12^

### Statistical analysis

In this sub-study, only participants who completed trial medication were included (per-protocol analysis) as prespecified in the trial protocol paper.^12^ A per-protocol analysis was chosen, as this is an exploratory sub-study, and the main interest was in participants who completed the study medication rather than the intention-to-treat population. Dual-echo pCASL and DP-pCASL MRI analyses were performed at the group level by comparing the follow-up results between groups with baseline results, age, and sex as covariates in individual analysis of covariance (ANCOVA) models. ANCOVA model assumptions were verified if a statistically significant difference between groups was found. A two-sided *P*-value < 0.05 was considered statistically significant. Unless otherwise specified, all results in text and tables are reported as mean ± standard error of the mean (SEM). We did not correct the analysis for multiple testing, and missing data were not imputed. Preprocessing and pipelining were performed in Python 3.10.8 using Advanced Normalization Tools 2.5.0 and FMRIB Software Library 6.0.7 for registration and segmentation and nilearn 0.11.1 for GLM-fitting. Data extraction and collation were performed in MATLAB R2022b, and statistical analyses were performed in RStudio 4.3.

## Results

**Figure 2:**
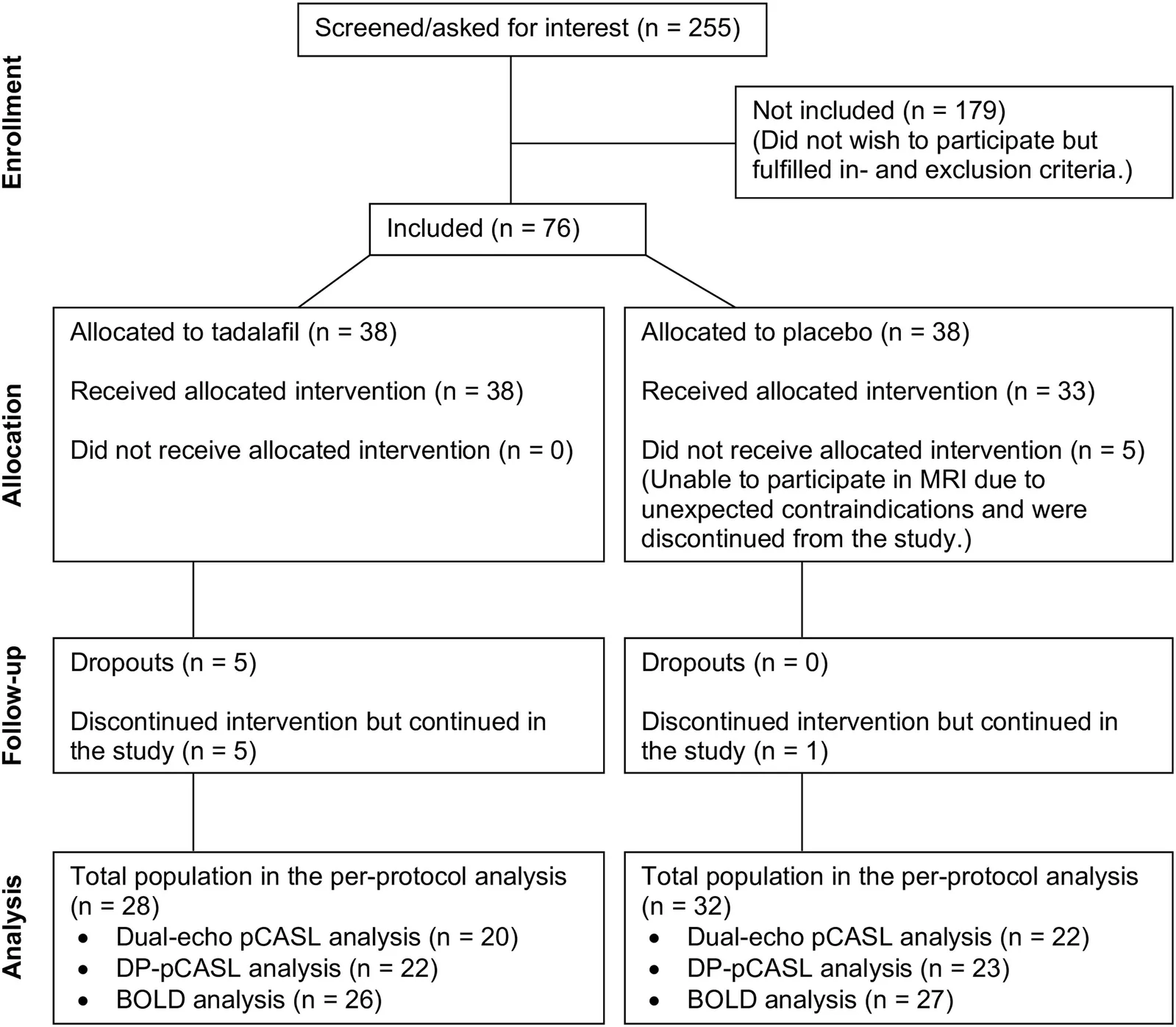
**Flowchart of included participants.** BOLD = blood-oxygen-level-dependent; DP-pCASL = diffusion-prepared pseudo-continuous arterial spin labelling; pCASL = pseudo-continuous arterial spin labelling.

In total, 76 participants were included in the ETLAS-2 trial, of whom 38 participants were allocated to tadalafil and 38 participants to placebo. In this per-protocol sub-study analysis, all participants who dropped out of the study or stopped trial medication prematurely were excluded. This resulted in 28 participants in the tadalafil group and 32 in the placebo group (Fig. 2; Table 1). Medication compliance rate was ≥90% in 26 of 28 participants in the tadalafil group and 31 of 32 in the placebo group.^10^ After removing 14 participants with severe ATT artefacts in the quality control of dual-echo pCASL scans and 4 participants who did not complete the dual-echo pCASL scans, 20 with tadalafil and 22 with placebo were included in the analysis. Both treatment groups were similar in baseline characteristics and baseline CBF in the pCASL population (Supplementary Table 1). In the event-related BOLD analysis, 26 participants with tadalafil and 27 with placebo were included, as 3 did not complete the scan and 4 scans were lost due to clerical errors. DP-pCASL scan was performed at the end of the MRI session. Due to the long scanning time (approximately 1 h and 20 min in total), the DP-pCASL scans were only conducted if the participants agreed to continue. This resulted in 22 participants receiving tadalafil and 23 placebo in the DP-pCASL scan for ATT analysis, as 15 participants did not complete this scan (Fig. 2). Participant recruitment stopped prematurely due to limited time in the funded period.

**Table 1:** Characteristics of per-protocol participants in the ETLAS-2 study

Characteristic	Tadalafil, *N* = 28 ^a^	Placebo, *N* = 32 ^a^	*P*-value ^b^
Age	68 (60, 72)	66 (62, 75)	0.8
Female	7 (25%)	7 (22%)	>0.9
Hypertension	22 (79%)	23 (72%)	0.8
Type 2 diabetes	3 (11%)	4 (13%)	>0.9
Hypercholesterolemia	27 (96%)	32 (100%)	0.5
Smoking status			0.8
Never	8 (29%)	10 (31%)	
Current	5 (18%)	8 (25%)	
Previous	15 (54%)	14 (44%)	
Ischaemic stroke	20 (71%)	21 (66%)	0.8
Transient ischemic attack	8 (29%)	11 (34%)	0.8
Periventricular WMH during admittance			0.5
Fazekas 0	2 (7.1%)	1 (3.4%)	
Fazekas 1	9 (32%)	15 (52%)	
Fazekas 2	12 (43%)	9 (31%)	
Fazekas 3	5 (18%)	4 (14%)	
*NA*	0	3	
Deep WMH during admittance			0.8
Fazekas 0	2 (7.1%)	3 (10%)	
Fazekas 1	10 (36%)	13 (45%)	
Fazekas 2	11 (39%)	9 (31%)	
Fazekas 3	5 (18%)	4 (14%)	
*NA*	0	3	

NA = not applicable; WMH = white matter hyperintensity.

^a^Median (Q1, Q3); *n* (%).

^b^Wilcoxon rank sum test; Fisher's exact test.

WMH scores are from the admission time point at the stroke unit.

### Cerebral blood flow

**Figure 3:**
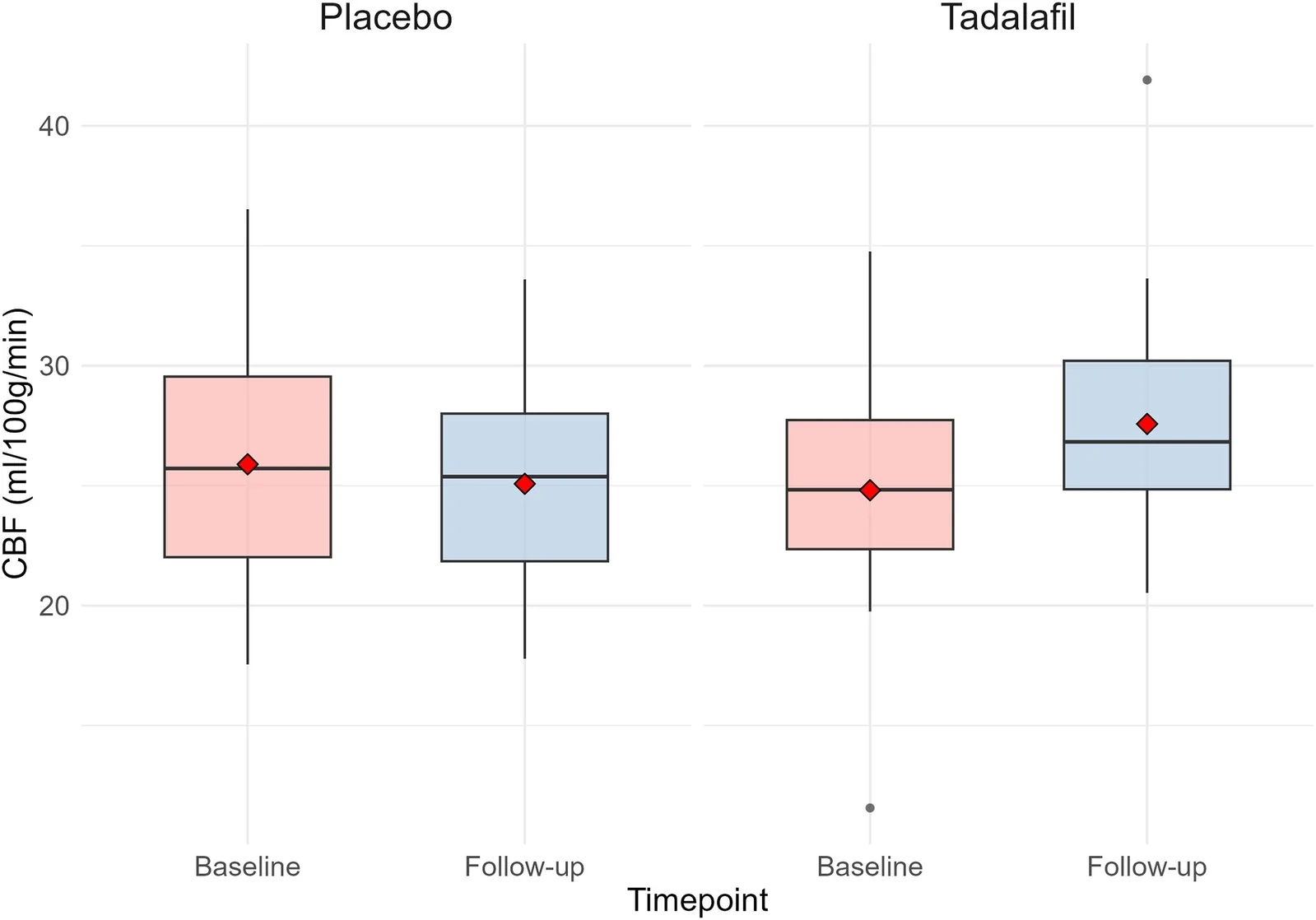
**CBF (ml/100 g/min) at baseline and follow-up between groups.** Boxplots show median, interquartile range (IQR), range 1.5 IQR as whiskers, and mean value as a red diamond. The two dots represent individual data points >1.5 IQR from the median. The adjusted mean group difference in whole-brain CBF at follow-up (ANCOVA adjusted for baseline CBF, age, and sex) was 3.33 ± 0.97 ml/100 g/min in favour of tadalafil compared with placebo (*P* = 0.001). Sample sizes were placebo (*N* = 22) and tadalafil (*N* = 20). CBF = cerebral blood flow.

**Figure 4:**
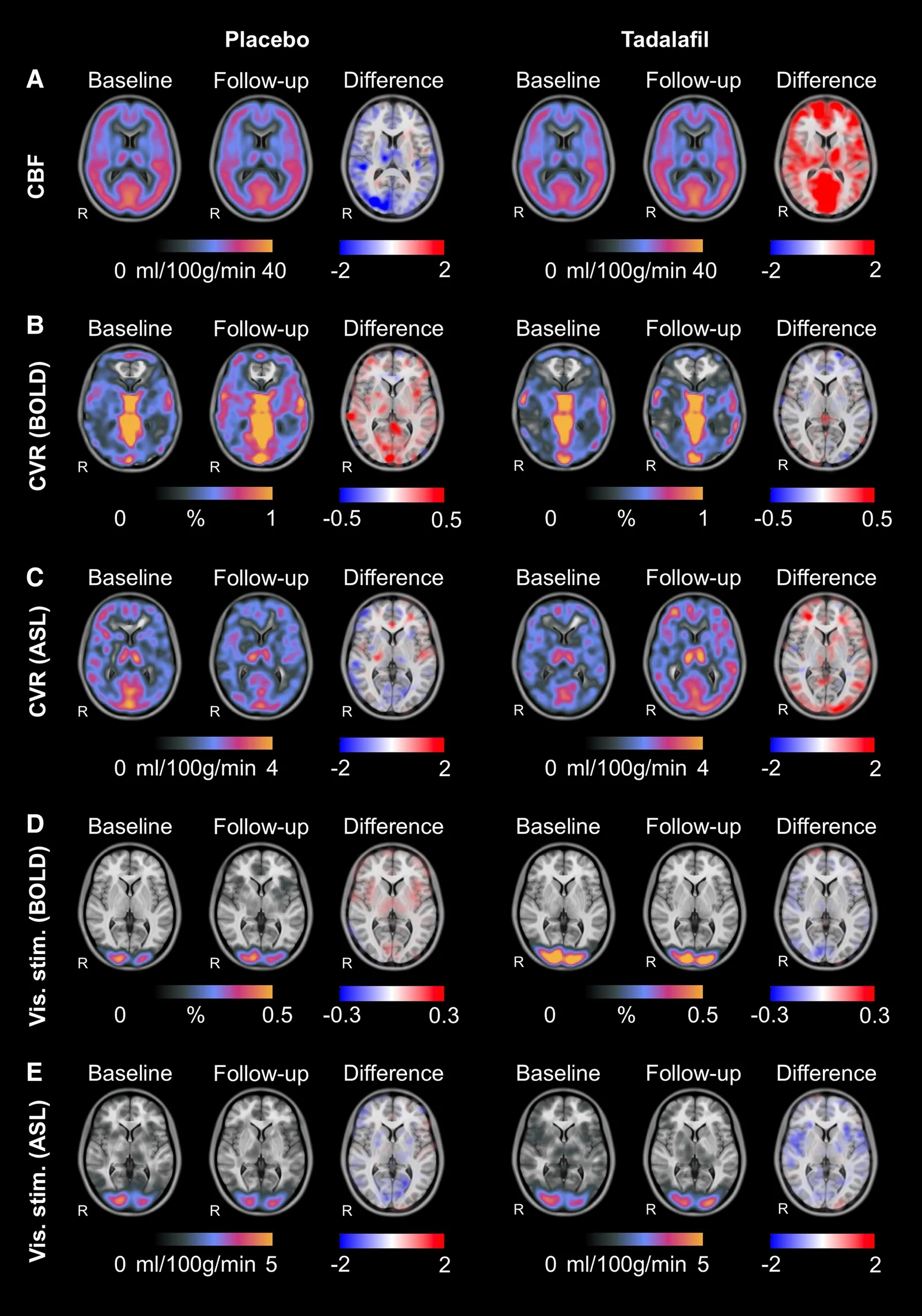
**CBF, CVR, and visual stimulation maps at baseline and follow-up between groups.** The maps display the average CBF (**A**), CVR (B, C), and visual stimulation response (D, E) in both groups at baseline and follow-up. The difference maps show the delta map (follow-up minus baseline) for both groups. The adjusted mean group differences at follow-up (ANCOVA adjusted for baseline values, age, and sex) were: (**A**) whole-brain CBF, 3.33 ± 0.97 ml/100 g/min (*P* = 0.001); (**B**) whole-brain CVR (BOLD), −0.08 ± 0.13% (*P* = 0.55); (**C**) whole-brain CVR (ASL), 0.54 ± 0.52 ml/100 g/min (*P* = 0.31); (**D**) visual stimulation (BOLD), −0.04 ± 0.09% (*P* = 0.69); and (**E**) visual stimulation (ASL), 0.80 ± 0.80 ml/100 g/min (*P* = 0.33). Positive values indicate higher values in the tadalafil group compared with placebo. Sample sizes were placebo (*N* = 22) and tadalafil (*N* = 20). ASL = arterial spin labelling; BOLD = blood-oxygen-level-dependent; CBF = cerebral blood flow; CVR = cerebrovascular reactivity; R = right; Vis. stim. = visual stimulation.

The mean (± SD) whole-brain CBF at baseline was 25.38 ± 5.11 ml/100 g/min in the total population. There was a treatment effect of tadalafil at follow-up with a mean CBF group difference of 3.33 ± 0.97 ml/100 g/min [95% CI: 1.37, 5.30, *P* = 0.001] in the tadalafil group compared to placebo (Figs 3 and 4, Table 2). A post-hoc paired *t*-test of CBF at baseline and follow-up within groups showed a mean increase of 2.76 ± 1.59 ml/100 g/min (*P* = 0.09) in the tadalafil group and −0.81 ± 0.55 ml/100 g/min (*P* = 0.585) in the placebo group. A second *post hoc* analysis was done after removing two outliers in the tadalafil group (Fig. 3), resulting in a mean difference of 2.79 ± 0.90 ml/100 g/min (*P* = 0.003) between groups.

**Table 2:** Predefined neurovascular outcomes in the ETLAS-2 study

Outcome	Baseline		Follow-up		Mean difference ± SEM a	
	Tadalafil *N* = 20^b^	Placebo *N* = 22^b^	Tadalafil *N* = 20^b^	Placebo *N* = 22^b^		*P*-value
Whole-brain: CBF (ml/100 g/min)	24.83 (22.14, 27.75)	25.72 (21.63, 30.00)	26.83 (24.51, 30.57)	25.38 (21.76, 28.23)	3.33 ± 0.97	0.001
Whole-brain: CBF hypercapnia response (ml/100 g/min)	1.69 (1.06, 2.36)	2.07 (1.05, 3.12)	2.09 (1.53, 3.41)	2.51 (1.09, 3.13)	0.54 ± 0.52	0.31
Whole-brain: CBF hypercapnia response (%)	6.76 (4.80, 11.77)	9.18 (3.72, 13.54)	7.94 (6.09, 12.01)	9.09 (5.67, 13.29)	0.49 ± 1.79	0.79
Whole-brain: BOLD hypercapnia response (%)	0.49 (0.33, 0.63)	0.55 (0.39, 0.69)	0.68 (0.24, 0.87)	0.63 (0.44, 0.76)	−0.08 ± 0.13	0.55
Visual ROI: CBF visual response (ml/100 g/min)	10.01 (9.20, 12.54) (*n* = 18)	10.58 (8.83, 11.78) (*n* = 21)	11.91 (9.58, 13.40) (*n* = 19)	10.05 (8.48, 13.35) (*n* = 18)	0.80 ± 0.80	0.33
Visual ROI: CBF visual response (%)	30.07 (26.34, 35.74) (*n* = 18)	28.20 (22.20, 36.08) (*n* = 21)	30.30 (27.30, 36.56) (*n* = 19)	29.40 (25.61, 35.74) (*n* = 18)	0.98 ± 2.54	0.70
Visual ROI: BOLD visual response (%)	1.17 (0.98, 1.39) (*n* = 18)	1.22 (0.90, 1.39) (*n* = 21)	1.08 (0.98, 1.35) (*n* = 19)	1.19 (0.94, 1.50) (*n* = 18)	−0.04 ± 0.09	0.69
Visual ROI: CMRO_2_ visual response (%)	13.65 (8.88, 16.35) (*n* = 18)	13.44 (9.12, 18.26) (*n* = 21)	11.70 (8.48, 19.31) (*n* = 18)	13.85 (9.88, 17.70) (*n* = 18)	−0.88 ± 2.74	0.75
Visual ROI: NVC	2.17 (1.67, 2.84) (*n* = 18)	2.01 (1.84, 2.32) (*n* = 21)	2.32 (1.41, 3.35) (*n* = 18)	2.04 (1.64, 2.21) (*n* = 18)	−7.49 ± 5.43	0.18
Whole-brain: ATT (s)	1.98 (1.91, 2.03) (*n* = 22)	1.93 (1.83, 2.01) (*n* = 23)	1.97 (1.89, 2.01) (*n* = 22)	1.92 (1.85, 2.01) (*n* = 23)	0.01 ± 0.03	0.86
Peak BOLD amplitude S1 cortex	0.68 (0.51, 0.92) (*n* = 24)	0.51 (0.39, 0.71) (*n* = 27)	0.67 (0.42, 0.82) (*n* = 26)	0.53 (0.39, 0.68) (*n* = 27)	−0.11 ± 0.12	0.36

ATT = arterial transit time; BOLD = blood-oxygen-level-dependent; CBF = cerebral blood flow; CMRO_2_ = cerebral metabolic rate of oxygen; NVC = neurovascular coupling; ROI = region of interest.

Visual ROI represents visually activated pixels in the occipital cortex (*t*-stat ≥ 5.1). Peak BOLD amplitude in the S1 cortex is from the fMRI BOLD scan and ATT from DP-pCASL; other outcomes are from dual-echo pCASL.

^a^Output from the ANCOVA analysis presenting the mean difference ± SEM between tadalafil and placebo at follow-up adjusted for baseline score, age, and sex.

^b^Median (*Q*1, *Q*3).

### Cerebrovascular reactivity, visual stimulation response, and neurovascular coupling

There were no differences between groups at follow-up for CBF and BOLD to hypercapnia (5% CO_2_), CBF, BOLD, and CRMO_2_ to visual stimulation, and NVC (Table 2). There were significant outliers in NVC, and in a post-hoc analysis of NVC results, the mean difference between groups at follow-up was 0.84 ± 0.38 (*P* = 0.037) after removing outliers defined as >1 SD. However, the overall model was not significant (*P* = 0.23).

### Arterial transit time and blood-brain-barrier water exchange

There were no differences between groups at follow-up for ATT (Table 2). Due to long ATTs in the patient population, results from the BBB water exchange calculation were either inconsistent or undefined; we have therefore excluded these from this report (see discussion).

### fMRI BOLD response to somatosensory stimulation

**Figure 5:**
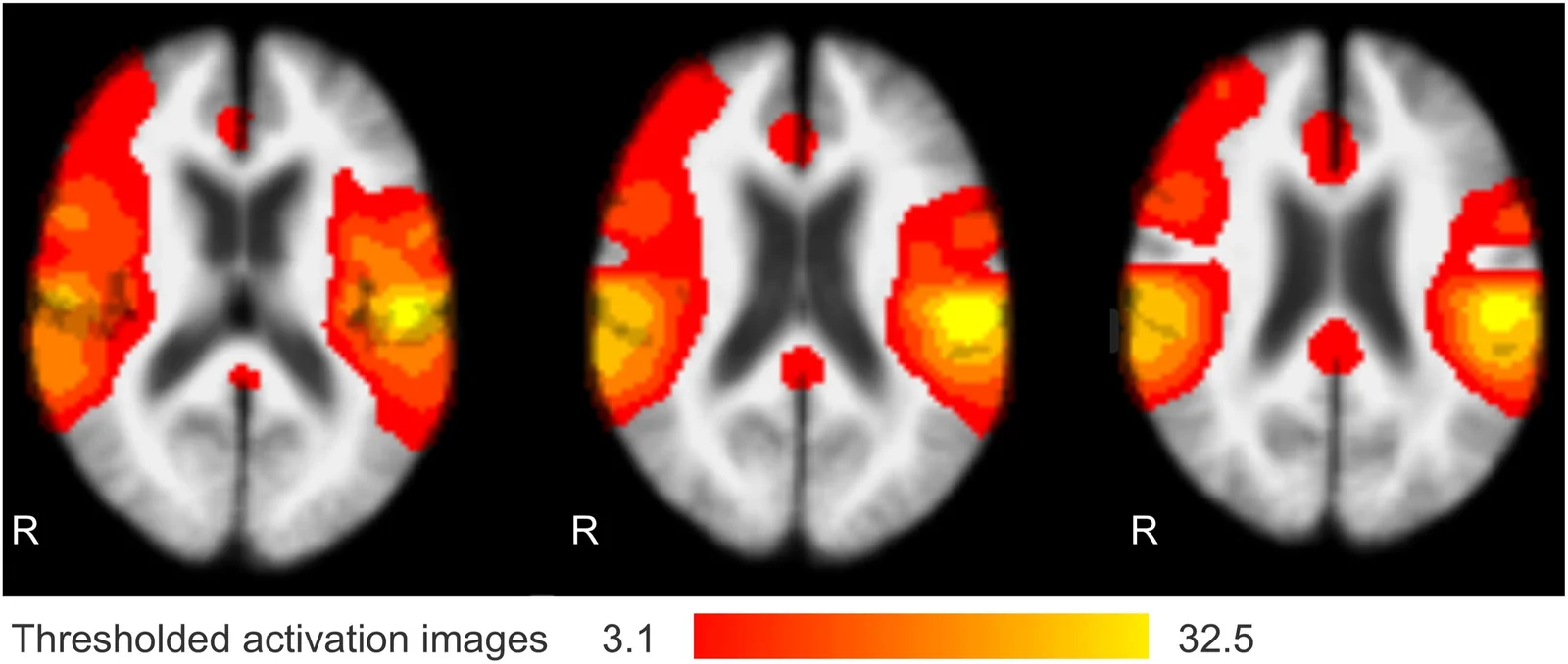
**Z-map of average activation across all participants from sensory index-finger stimulation in fMRI BOLD scan.** The highest BOLD response is observed in the left S2 somatosensory cortex, with a significant response also present in the contralateral S2. Z ≥ 3.1, cluster significance threshold *P* = 0.05 (corrected). The sample size for this average activation map across all participants was 53. BOLD = blood-oxygen-level-dependent; R = right.

The mean index-finger stimulation at baseline in the tadalafil and placebo groups was 12.2 ± 3.2 mA and 12.6 ± 3.2 mA, respectively. At follow-up, stimulation in the tadalafil group was 12.6 ± 2.7 mA, and in the placebo group, 12.3 ± 2.4 mA. There was no difference in stimulation strength from baseline to follow-up (*P* = 0.75). The stimulation provided a significant BOLD response in relevant anatomical somatosensory areas (Fig. 5). However, a statistical map of the BOLD response (from FSL FEAT) showed no difference between treatment groups. There was no difference between groups in the mean peak BOLD activation amplitude at the S1 somatosensory cortex (0.11 ± 0.12, *P* = 0.36).

## Discussion

In this MRI sub-study, we examined the neurovascular outcomes from the ETLAS-2 trial. We found that tadalafil increased whole-brain CBF significantly compared to placebo, with a mean difference of 3.33 ± 0.97 ml/100 g/min between groups. For the total population, the average whole-brain CBF at baseline was 25.38 ± 5.11 ml/100 g/min, which is reduced compared to the normal values of ∼40 ml/100 g/min in healthy adults.^17^ The tadalafil-induced CBF changes represented a 13% increase in CBF. There were no differences between groups regarding CVR, visual-induced CBF changes, neurovascular coupling index, ATT, and event-related BOLD to sensory index-finger stimulation. Previously published results from the ETLAS-2 trial showed a trend towards a reduction in WMH volume following tadalafil; however, participants exposed to the dose of 20 mg daily reported side effects which reduced compliance compared to placebo.^10^ Furthermore, 3 months of tadalafil did not improve cognitive functioning.^11^

Cerebrovascular functioning, including CBF and CVR, may inform on the severity of CSVD since multiple studies have found reduced CBF in persons with CSVD compared to no evident CSVD using methods such as positron emission tomography (PET), single-photon emission computerized tomography (SPECT), MRI (arterial spin labelling, phase-contrast, and dynamic-contrast enhanced (DCE)), and transcranial Doppler (TCD).^1,5^ These methods often build on different assumptions and models and cannot always be directly compared. However, a good correlation has been shown between CBF measurements using ^15^O-H_2_O-PET (current gold standard) and pCASL-MRI.^17^ The PASTIS trial testing a single dose of tadalafil versus placebo in a CSVD cohort using pCASL MRI (3T Achieva scanner, Philips) found a mean grey matter CBF of 33 ml/100 g/min,^18^ and whole-brain CBF of 25–27 ml/100 g/min^19^ comparable to our whole-brain CBF measurements of 25 ml/100 g/min that included both grey and white matter. In the PASTIS trial, CBF was mapped in grey matter, white matter, and deep grey matter, but they found no effect of single-dose tadalafil versus placebo on resting CBF in either brain region.^18^ Further, they did not find an effect of tadalafil on any cognitive outcome, although there was a trend towards improved Digit Span Forward score following tadalafil.^20^ In the OxHARP study, where 3 weeks of daily sildenafil was tested against placebo or cilostazol in patients with CSVD, there was evidence of improved cerebrovascular function after sildenafil.^3^ Sildenafil 50 mg, taken three times daily, significantly improved CBF in white matter, WMH, cortical and subcortical grey matter, and brainstem.^3^ In the OxHARP study, cortical grey matter CBF increased by 3.69 ml/100 g/min after sildenafil, consistent with our results for tadalafil.^3^ Besides increased CBF, they also found increased CVR by TCD and MRI with hypercapnia, but no changes in cerebral pulsatility with TCD. In the OxHARP study, MRI perfusion imaging was acquired using a pCASL sequence, whereas CVR measurements were performed separately during hypercapnia using a BOLD readout.^3^ The BOLD-based CVR method may be more robust and less prone to artefacts than the dual-echo pCASL approach, which could partly explain why the OxHARP trial observed increased CVR in response to sildenafil, in contrast to our findings. The dual-echo pCASL sequence used in our trial was optimized for cortical BOLD and CBF measurements. Consequently, CBF was not quantified in white matter or subcortical grey matter regions, in contrast to the PASTIS and OxHARP trials. A systematic review of PDE-5 inhibition on CBF concluded that there is no evident effect of PDE-5 inhibition on resting CBF in humans.^21^ These findings are in contrast to our results and the OxHARP trial results and may reflect the limited research using PDE-5 inhibitors in CSVD and endothelial dysfunction. The systematic review found evidence of enhanced CVR following PDE-5 inhibitors, consistent with the OxHARP trial; however, this finding was not supported by current findings from the ETLAS-2 trial.^21^

In the study of CSVD, CVR and NVC are intriguing factors, as they indicate how blood vessels can enhance blood flow in response to vasodilatory stimuli and adjust local perfusion based on neuronal activity, respectively. Studies show an association between increasing CSVD burden and lower CVR.^6^ In a cross-sectional study, a reduced CVR was associated with increasing CSVD burden, and this was also linked to reduced cognition.^7^ Further, lower CVR has also been shown to predict an increasing amount of WMH in longitudinal data, highlighting that reduced vascular reactivity may contribute to the development of WMH rather than resulting from them.^22^ Similarly, reduced NVC, measured with different modalities, has also been associated with increasing severity of CSVD markers.^23^ CVR and NVC can potentially serve as markers for CSVD in conjunction with CBF. Although the setup of these measurements can be demanding, they may provide valuable insights into the disease.

The strength of this ETLAS-2 sub-study was that the trial was randomized, double-blind, placebo-controlled, and we were able to measure cerebrovascular parameters without using a contrast agent or exposing participants to radiation. The MRI scan included the acquisition of CBF, CVR, visual stimulation, NVC, and ATT, together with event-related BOLD, which extensively evaluated the effect of tadalafil on cerebrovascular functioning. All MRI scans were done on the same scanner, with the same examiners ensuring consistency throughout the scans.

The study's limitations include not reaching the planned sample size and the need to exclude many participants. Ten individuals stopped taking tadalafil prematurely due to side effects, resulting in a smaller study population than anticipated. For future trials, a lower dose of tadalafil or a dose-escalation approach could be used to enhance treatment compliance. Additionally, we did not validate the dual-echo pCASL sequence combined with the applied MRI modelling approach in a larger sample of healthy individuals. Consequently, the observed whole-brain CBF values may be biased towards lower values and thus underestimated relative to true physiological values. In total, 18 participants were excluded from the dual-echo pCASL analysis. Of these, 4 were excluded for not completing the scan, and 14 individuals, particularly elderly participants, were excluded due to severe ATT artefacts, despite using a prolonged post-labelling delay of 2 s compared to the usual 1.6 s. Thus, this study cannot verify the effect of tadalafil in the most elderly participants, which is a limitation since old age is an independent risk factor for developing CSVD.^8^ The participants who were excluded from the dual-echo pCASL analysis did not differ in any other baseline characteristics (Supplementary Table 2). The long ATT was also reflected in our measurements: average ATT for the total population was 1.95 s at baseline compared to 1.4–1.5 s using a similar method in healthy controls.^15^ This finding is consistent with previous reports showing prolonged arterial transit time in CSVD.^5^ The extended ATT violated the assumptions required by the BBB water exchange model,^15^ namely that all blood water had to be delivered to the tissue, which led to inconsistent results from the model fitting. Due to the long scanning time, not all participants performed the DP-pCASL scan, resulting in missing data on some participants. Additionally, the NVC results were influenced by several outliers. Therefore, a *post hoc* sensitivity analysis excluding values beyond 1 standard deviation was performed for this outcome. Although this *post hoc* test did show a group difference, the overall statistical model was non-significant and therefore inconclusive. For future trials on cerebrovascular function in CSVD, a more simplistic MRI setup less prone to artefacts would be preferable, including plain pCASL for CBF, BOLD response to hypercapnia for CVR, and DCE MRI for BBB measurements. However, a recent study successfully measured BBB water exchange in different CSVD subtypes, including sporadic CSVD.^24^ They applied a modified version of the two-stage approach,^25^ which uses a 3D gradient and spin echo (GRASE) readout rather than EPI in the original sequence.^15^ GRASE readout has been speculated to result in shorter estimated ATT,^25^ which may be necessary for model fitting in this patient population.

This study showed that 3 months of pharmacological PDE-5 inhibition with tadalafil increased resting CBF compared to placebo in patients with CSVD. This finding concurs with the reported results of daily sildenafil treatment.^3^ Together, these studies provide evidence that CSVD-related vascular impairment can be pharmacologically reversed to some degree. Yet, it remains to be clarified whether a 13% increase in CBF translates into a clinically meaningful benefit under long-term treatment. Continuous treatment for 1 year with the nitric oxide donor, isosorbide mononitrate, improved cognition in patients with CSVD and reduced recurrent stroke risk compared to no additional treatment.^26^ Isosorbide mononitrate affects the NO—cGMP pathway upstream of cGMP, thereby influencing the same molecular pathway. This warrants an extended treatment period with PDE-5 inhibitors in CSVD to explore the possible clinical implications.

In conclusion, we found a low baseline whole-brain CBF of 25.4 ml/100 g/min and tadalafil increased CBF by 3.3 ml/100 g/min, representing a 13% CBF increase. We hypothesized that tadalafil would improve on-demand vascular function, as in the treatment of erectile dysfunction, measured by neurovascular coupling index and event-related BOLD response. However, we found no effect of tadalafil on CVR, visual stimulation response, neurovascular coupling, or event-related BOLD. This may be due to the limited number of participants in the study, which was not powered for these outcomes. Since our improved CBF result was observed in a subgroup of a smaller trial, we propose that the findings be replicated in a trial with a lower tadalafil dose or a dose-escalation approach over a longer treatment period. This would help to clarify if tadalafil improves neurovascular function and thus lowers recurrent stroke and dementia risk.

## Supplementary Material

fcag309_Supplementary_Data

## Data Availability

The data that support the findings of this study are available from the corresponding author upon reasonable request. Python and MATLAB scripts used for MRI processing and data collection are available at: https://doi.org/10.5281/zenodo.19233779.
