# Supplementary material for: Neurovascular effects of tadalafil in patients with cerebral small vessel disease: ETLAS-2 substudy
Source: Brain Commun. 2026 Aug 13;8(4):fcag309. doi: 10.1093/braincomms/fcag309 (PMC13504703; doi:10.1093/braincomms/fcag309)
Supplement: Supplementary file 1 [file fcag309_supplementary_data.docx]

**Supplementary Table 1 Characteristics of pCASL participants in the ETLAS-2 Study**

| **Characteristic** | **Tadalafil,  *N* = 20^1^** | **Placebo,  *N* = 22^1^** | ***P*-value^2^** |
| --- | --- | --- | --- |
| Age | 63 (58, 70) | 65 (58, 69) | 0.6 |
| Female | 5 (25%) | 5 (23%) | >0.9 |
| Hypertension | 16 (80%) | 15 (68%) | 0.5 |
| Type 2 diabetes | 2 (10%) | 4 (18%) | 0.7 |
| Hypercholesterolemia | 19 (95%) | 22 (100%) | 0.5 |
| Smoking status |  |  | >0.9 |
| Never | 5 (25%) | 7 (32%) |  |
| Current | 3 (15%) | 4 (18%) |  |
| Previous | 12 (60%) | 11 (50%) |  |
| Ischemic stroke | 16 (80%) | 15 (68%) | 0.5 |
| Transient Ischemic Attack | 4 (20%) | 7 (32%) | 0.5 |
| Periventricular WMH during admittance |  |  | 0.2 |
| Fazekas 0 | 2 (10%) | 1 (5.3%) |  |
| Fazekas 1 | 6 (30%) | 12 (63%) |  |
| Fazekas 2 | 9 (45%) | 5 (26%) |  |
| Fazekas 3 | 3 (15%) | 1 (5.3%) |  |
| *NA* | 0 | 3 |  |
| Deep WMH during admittance |  |  | 0.3 |
| Fazekas 0 | 2 (10%) | 3 (16%) |  |
| Fazekas 1 | 7 (35%) | 11 (58%) |  |
| Fazekas 2 | 8 (40%) | 4 (21%) |  |
| Fazekas 3 | 3 (15%) | 1 (5.3%) |  |
| *NA* | 0 | 3 |  |
| Baseline CBF | 24.8 (22.1, 27.7) | 25.7 (21.6, 30.0) | 0.7 |

^1^Median (Q1, Q3); n (%)

^2^Wilcoxon rank sum test; Fisher's exact test.

NA = not applicable; WMH = white matter hyperintensity.

WMH scores are from the admission time point at the stroke unit.

**Supplementary Table 2 Characteristics of participants that were included/excluded in the pCASL analysis in the ETLAS-2 Study**

| **Characteristic** | **Included  N = 42^1^** | **Excluded  N = 18^1^** | ***P*-value^2^** |
| --- | --- | --- | --- |
| Treatment |  |  | >0.9 |
| Placebo | 22 (52%) | 10 (56%) |  |
| Tadalafil | 20 (48%) | 8 (44%) |  |
| Age | 65 (58, 69) | 72 (64, 80) | <0.001 |
| Female | 10 (24%) | 4 (22%) | >0.9 |
| Hypertension | 31 (74%) | 14 (78%) | >0.9 |
| Type 2 diabetes | 6 (14%) | 1 (5.6%) | 0.7 |
| Hypercholesterolemia | 41 (98%) | 18 (100%) | >0.9 |
| Smoking status |  |  | 0.2 |
| Never | 12 (29%) | 6 (33%) |  |
| Current | 7 (17%) | 6 (33%) |  |
| Previous | 23 (55%) | 6 (33%) |  |
| Ischemic stroke | 31 (74%) | 10 (56%) | 0.2 |
| Transient Ischemic Attack | 11 (26%) | 8 (44%) | 0.2 |
| Periventricular WMH during admittance |  |  | 0.3 |
| Fazekas 0 | 3 (7.7%) | 0 (0%) |  |
| Fazekas 1 | 18 (46%) | 6 (33%) |  |
| Fazekas 2 | 14 (36%) | 7 (39%) |  |
| Fazekas 3 | 4 (10%) | 5 (28%) |  |
| *NA* | 3 | 0 |  |
| Deep WMH during admittance |  |  | 0.10 |
| Fazekas 0 | 5 (13%) | 0 (0%) |  |
| Fazekas 1 | 18 (46%) | 5 (28%) |  |
| Fazekas 2 | 12 (31%) | 8 (44%) |  |
| Fazekas 3 | 4 (10%) | 5 (28%) |  |
| *NA* | 3 | 0 |  |

^1^Median (Q1, Q3); n (%)

^2^Wilcoxon rank sum exact test; Fisher's exact test; Wilcoxon rank sum test

NA = not applicable; WMH = white matter hyperintensity.

WMH scores are from the admission time point at the stroke unit. Fourteen of the excluded participants were excluded due to severe arterial transit time artefacts, while the other 4 due to not completing the pCASL scan.
